# Evaluation of patient-reported outcomes in over 10,000 patients using pneumatic compression therapy for lower-extremity lymphedema

**DOI:** 10.1016/j.jvsv.2026.102548

**Published:** 2026-06-02

**Authors:** Ashna Raiker, Alisha Raiker, Steven Dean, Antonios Gasparis, Nicos Labropoulos

**Affiliations:** a Department of Vascular Surgery, The Mount Sinai Hospital, New York, NY; b Stony Brook University, New York, NY; c Division of Cardiovascular Medicine, Ohio State University Wexner Medical Center, Columbus, OH; d Division of Vascular Surgery, Northwell Health, Huntington, NY; e Department of Surgery, Stony Brook Medicine, Stony Brook, NY

**Keywords:** Lymphedema, Pneumatic compression device, Patient reported outcomes, Patient satisfaction, Treatment compliance

## Abstract

**Objective:**

Lymphedema treatment varies from compression and manual lymphatic drainage to surgical measures. Although compression demonstrates clinical benefit, poor adherence and dissatisfaction can contribute to treatment failure. This study aimed to assess patient-reported satisfaction, device usage, and symptom improvement with pneumatic compression device therapy.

**Methods:**

This online survey-based study was conducted from January 1, 2024, to December 31, 2024, with 35,522 invitations delivered without incentive to respond. Patients with lower-extremity edema who had been prescribed a medical pneumatic compression device and were included within the company database were eligible for inclusion. Patients with multiple or different devices over the study time period were excluded. Surveys were administered in English or Spanish at 45 and 90 days, encompassing device satisfaction, device usage, and symptom improvement based on a predetermined list of symptoms. Questions were graded on a Likert scale of 1 to 5, with 5 representing complete agreement or extreme satisfaction.

**Results:**

After exclusions, survey data from 10,543 patients (30%) were analyzed. Participants had a mean age of 69.8 ± 11.8 years, 64% were female, and 92% had bilateral lymphedema. At 45 days, patients reported high device satisfaction in all categories: overall satisfaction, ease of use, functionality, quality, and reliability. Satisfaction across all categories improved as device frequency increased from less than once a week (48%-77%) to 1 to 2 d/wk (70%-91%) to daily (85%-94%) for Likert 4 and 5 (*P* < .0001). Furthermore, patient-reported symptoms and ability to perform activities improved with increased device usage as well from less than once a week (34%), to 1 to 2 d/wk (53%), to everyday use (67%) for Likert 4 and 5 (*P* < .0001). There was minimal additional benefit for both satisfaction and symptom improvement, when comparing 3 to 6 d/wk vs daily use. With daily use, most reported improvement in swelling (58%), heaviness (34%), and pain (25%). Comparing 45- and 90-day time points, across all satisfaction categories, the responses stayed the same for 85% to 92% of patients (73%-88% of which were already Likert 4-5), decreased for 4% to 8%, and increased for 3% to 7%. For device usage, responses stayed the same for 74%, decreased for 19%, and increased for 7%. For symptom improvement, responses stayed the same for 78% (56% of which were already Likert 4-5), decreased for 10%, and increased for 13%.

**Conclusions:**

Patient-reported satisfaction and symptom improvement with pneumatic compression therapy was high and improved with higher device usage. The highest improvement was in reduction in swelling, heaviness, and pain. Outcomes remained stable comparing 45 to 90 days.

**Article Highlights**

• **Type of Research:** Retrospective observational study
• **Key Findings:** A survey of 10,543 patients receiving pneumatic compression device therapy for lower-extremity lymphedema demonstrated high patient-reported satisfaction (85%-94%) and symptom improvement (67%) with daily use. Clinical benefit was associated with increased device usage and was stable over time. Comparable outcomes with 3 to 6 d/wk vs daily use support its practicality.
• **Take Home Message:** Pneumatic compression device therapy is a clinically beneficial treatment modality with high patient-reported satisfaction and symptom improvement warranting significant incorporation into treatment regimens for lower-extremity lymphedema.

Lymphedema is an incurable debilitating condition managed with multimodality treatments ranging from compression therapy and manual lymphatic drainage to surgery.^1^^,^^2^ It poses a significant burden on both patients and health care systems because of its longitudinal treatment course, innumerable hospitalizations, and high expenditure.^3^ Compression remains the cornerstone of lymphedema treatment and is a major component of the standard complex decongestive therapy.^2^

Pneumatic compression devices (PCDs) have emerged as an effective aid for the home maintenance phase of complex decongestive therapy and have evolved from single chamber versions to advanced iterations that use pressure modulation and sequential compressions to model manual lymphatic therapy.^2^^,^^4^ Although the literature on lower-extremity PCD use is limited, their use is recommended in select patient populations by the Lymphedema Framework and American Venous Forum clinical practice guidelines.^5^ A large prospective study demonstrated significant reductions in lower limb volumes in 90% of patients and improved quality of life.^4^ Another clinical trial further demonstrated success in reducing ulceration, cellulitis, hospital admissions, and resource usage.^3^ PCD success can be attributed to various mechanisms including reduction of venous reflux, improved calf-muscle contractility, and increased lymphatic propulsion.^4^ Patient nonadherence remains a major cause of treatment failure with adherence rates widely ranging from 28% to 69%.^6^ Uncomfortable devices, 1 hour duration of therapy, limitations on activities of daily living, financial constraints, and limited patient education are all contributing factors to low adherence and treatment failure.^6^

In recent times, the value of patient-reported outcomes to optimize treatment has become increasingly evident.^7^ Metrics to evaluate lymphedema treatment such as reasons for nonadherence, satisfaction, severity of symptoms (eg swelling and heaviness), ease of device use, and quality of life can often be subjective.^7^ Patient-reported data can reveal discrepancies between treatment expectations and progression and challenges in balancing cost and effectiveness and can identify inadequately addressed needs.^7^ However, there is currently a gap in patient-reported data for PCD use, which is limiting both the creation of optimal treatment plans and adequate insurance coverage.^3^^,^^8^

Thus, this retrospective study aimed to evaluate the clinical benefit of PCD therapy for lymphedema using patient-reported outcomes. Specifically, the study investigated the Tactile Medical PCDs (Flexitouch and Entre Plus) with respect to frequency of device usage, satisfaction, and symptom improvement.

## Methods

This study was conducted with survey design from January 1, 2024, to December 31, 2024, to evaluate patient usage, symptom improvement, and satisfaction with PCD therapy.

### Subject population

Patients who were prescribed a Tactile Medical PCD by an independent private health care provider in the United States were included in this study. Inclusion criteria consisted of patients with an email address recorded in the company customer relationship management system, an active territory account manager, and those who were shipped a Tactile Medical PCD in the previous 45 or 90 days.

### Data collection

Patients were sent a link through email to a descriptive survey managed on an online Medallia Experience Cloud platform. Medallia is an independent enterprise experience platform that helps companies capture and analyze patient feedback. Surveys were sent at both 45 and 90 days after the device shipment dates. If no response was received, a reminder email was sent 3 days later. Surveys were administered in English and Spanish, with English as the default and the survey window stayed active for 14 days post email.

### Patient health information /consent

Patient consent was captured when patients completed a consent form and provided their contact information. Patients reviewed the consent form which provided Tactile Medical's Notice of Privacy Practices and the Privacy Statement available on the company website. The Privacy Statement outlined how patient health information (PHI) is used and disclosed for research purposes: “We may use your information for research related to the products and services provided by Tactile Medical. For example, we may evaluate the number of patients using our products with a specific clinical diagnosis. If we use or disclose your information for research, your information will be de-identified to ensure you will not be identifiable.”

Patients were informed of their rights as well through these documents. Medallia's privacy policy was provided on the invitation and reminder emails. Patients could opt out of surveys at any time using the link in the email.

PHI was protected through several methods and was transmitted to Medallia through secure files. Medallia security is compliant with GDPR (General Data Protection Regulation), CCPA (California Consumer Privacy Act), HIPAA (Health Insurance Portability and Accountability Act), HITRUST (Health Information Trust Alliance), CBPR (Cross-Border Privacy Rules), and PRP (Privacy Recognition for Processors) certified. Medallia also holds SOC 2 Type II (System and Organization Controls 2 Type II) platform compliance. Data centers have common security practices including 24/7-manned guards, biometric access controls, and closed-circuit video. A business associate agreement was executed between Tactile Medical and Medallia.

### Survey instrument

The emailed survey was created at Tactile Medical based on the needs of the organization. The survey consisted of nine clinical questions with varying response types including, Likert scales 1 to 5, open text, single selection, and multiple selection.

The domains evaluated through the survey were satisfaction with the device (such as overall, ease of use, functionality, quality, and reliability), reported adherence to prescribed therapy, and reported symptom improvement.

### Statistical analyses

The analyses presented are generally exploratory in nature because there was no prespecified statistical analysis plan. Continuous variables are presented as means, standard deviations, and 95% confidence intervals. Categorical variables are presented as per-level counts, percentages, and 95% confidence intervals using the Wilson score method.

Associations between patient survey Likert (1-5) responses and device usage, for combined Likert values of 4 to 5 (favorable responses) vs 1 to 3 (unfavorable to neutral responses) as two derived groups, were assessed using *χ*^2^ tests. For patients with survey responses at both 45 and 90 days, comparisons of paired survey response changes between the two time points were assessed using nonparametric tests for the three derived ordinal values of decreased, no change, and increased.

Missing data were not imputed, and all results were reported on the available data only. Two-sided *P* values of <.05 are used to reject the null hypothesis. All data processing, summarization, and analyses were performed using R, version 4.4.3 (R Foundation for Statistical Computing) or higher.^9^ Data analysis was conducted independently and without any influence from the device manufacturer. No institutional review board approval was necessary because it was a survey-design study.

## Results

### Study group

**Fig 1:**
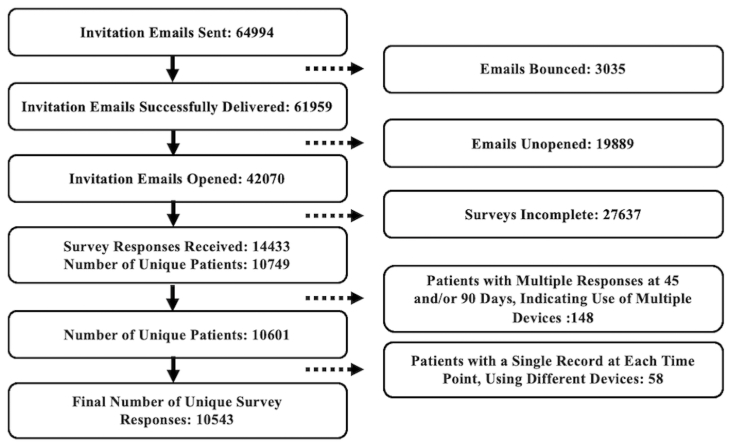
Flow diagram illustrating the process of survey dissemination to patients prescribed pneumatic compression devices, combined for both 45- and 90-day time points. The response rate of 30% was calculated as the number of survey responses divided by the number of invitations delivered to unique patients. The number of survey responses was 10,543, and the total number of invitations delivered across the 45- and 90-day time points was 64,994 invitations to 35,522 unique patients.

From the study range of January 1, 2024, to December 31, 2024, 35,522 surveys were successfully delivered, with 10,749 responses received. After exclusions, 10,543 patients (30%) were analyzed (Fig 1). Patients had a mean age of 69.8 ± 11.8 years, with 64% being female and 92% diagnosed with bilateral lymphedema.

### 45-Day outcomes

**Fig 2:**
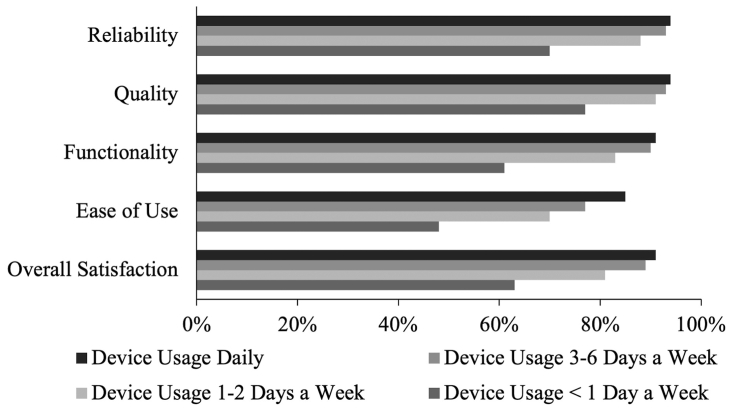
Change in patient-reported satisfaction across various categories (reliability, quality, functionality, ease of use, and overall satisfaction) with increasing frequency of device usage. Patient-reported satisfaction was measured as combined Likert scores of 4 and 5 (*P* < .0001) at the 45-day time point (n = 7524 patients).

Patients reported high satisfaction with the device overall, including its ease of use, functionality, quality, and reliability (Table I). Satisfaction was directly proportional to the frequency of device usage. Satisfaction across all categories rose as usage increased from less than once a week (48%-77%) to 1 to 2 d/w (70%-91%) to 3 to 6 d/wk (77%-93%) to daily (85%-94%) for a combined Likert score of 4 and 5 (*P* < .0001). Increasing usage from 3 to 6 d/wk to daily use offered minimal additional benefit overall (Fig 2).

**Table I:** Patient-reported satisfaction with pneumatic compression device use across various categories including reliability, quality, functionality, ease of use, and overall satisfaction measured on a Likert scale of 1 to 5 at the 45-day time point (n = 7552 patients)

Patient response summary	No. of patients
Overall satisfaction	
Mean ± SD (CI)	4.4 ± 0.9 (4.4-4.4)
Likert scale responses	
1	187 (2.5 [2.1-2.9])
2	179 (2.4 [2.1-2.7])
3	610 (8.1 [7.5-8.7])
4	1884 (24.9 [24.0-25.9])
5	4692 (62.1 [61.0-63.2])
Ease of use	
Mean ± SD (CI)	4.2 ± 1.0 (4.2-4.2)
Likert scale responses	
1	258 (3.4 [3.0-3.9])
2	315 (4.2 [3.7-4.6])
3	1007 (13.3 [12.6-14.1])
4	2050 (27.1 [26.2-28.2])
5	3922 (51.9 [50.8-53.1])
Functionality	
Mean ± SD (CI)	4.4 ± 0.9 (4.4-4.5)
Likert scale responses	
1	183 (2.4 [2.1-2.8])
2	157 (2.1 [1.8-2.4])
3	576 (7.6 [7.0-8.2])
4	1881 (24.9 [23.9-25.9])
5	4755 (63 [61.9-64.0])
Quality	
Mean ± SD (CI)	4.6 ± 0.8 (4.5-4.6)
Likert scale responses	
1	136 (1.8 [1.5-2.1])
2	105 (1.4 [1.1-1.7])
3	405 (5.4 [4.9-5.9])
4	1594 (21.1 [20.2-22.0])
5	5312 (70.3 [69.3-71.4])
Reliability	
Mean ± SD (CI)	4.6 ± 0.8 (4.6-4.6)
Likert scale responses	
1	153 (2.0 [1.7-2.4])
2	89 (1.2 [1.0-1.4])
3	432 (5.7 [5.2-6.3])
4	1502 (19.9 [19.0-20.8])
5	5376 (71.2 [70.2-72.2])

*CI*, Confidence interval (Wilson method); *SD*, standard deviation. Likert scale data are represented as the number of patients (% [95% CI]). This scale ranges from 1 (not at all satisfied) to 5 (extremely satisfied).

**Fig 3:**
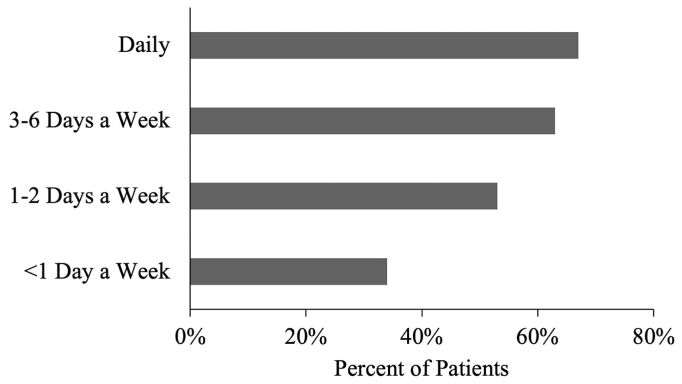
Change in symptom improvement and/or overall ability to perform normal activities with increasing frequency of device usage. Patient-reported satisfaction was measured as combined Likert scores of 4 and 5 (*P* < .0001) at the 45-day time point (n = 7430 patients).

Patients reported high levels of symptom improvement and ability to perform normal activities (Table II). It similarly improved with increased frequency of device usage from less than once a week (34%), to 1 to 2 d/wk (53%), 3 to 6 d/wk (63%) to everyday use (67%) for combined Likert score of 4 and 5, *P* < .0001. Increasing usage from 3 to 6 d/wk to daily use offered no real benefit (Fig 3). Among patients who used the device daily, most patients reported symptom improvement in swelling (58%), heaviness (34%), and pain (25%) (Table III). All three improved with device usage from less than once a week (22%, 12%, and 12%) to daily use (58%, 34%, and 25%), respectively (*P* < .0001).

**Table II:** Patient-reported symptom improvement and/or overall ability to perform normal activities with pneumatic compression device measured on a Likert scale of 1 to 5 at the 45-day time point (n = 7450 patients)

Patient response summary	No of patients
Mean ± SD (CI)	3.8 ± 1.2 (3.8-3.8)
Likert scale responses	
1	473 (6.3 [5.8-6.9])
2	427 (5.7 [5.2-6.3])
3	1887 (25.3 [24.4-26.3])
4	2087 (28.0 [27.0-29.0])
5	2576 (34.6 [33.5-35.7])

*CI*, Confidence interval (Wilson method); *SD*, standard deviation.

Likert scale data are represented as the number of patients (% [95% CI]). This scale ranges from 1 (not at all satisfied) to 5 (extremely satisfied).

**Table III:** Patient-reported improvement in various lower-extremity symptoms with pneumatic compression device therapy at the 45-day time point (n = 7552)

Symptom	No. of patients (% [95% CI])
Ability to walk longer distances	1276 (16.9 [16.1-17.8])
Ability to stand for longer periods of time	1450 (19.2 [18.3-20.1])
Self-image	715 (9.5 [8.8-10.1])
Less pain	1723 (22.8 [21.9-23.8])
Less heaviness	2341 (31.0 [30.0-32.1])
Less swelling	4019 (53.2 [52.1-54.3])
Sleep	655 (8.7 [8.1-9.3])
Compression garments easier to put on	1374 (18.2 [17.3-19.1])
Fit of clothing/jewelry	568 (7.5 [6.9-8.1])

*CI*, Confidence interval (Wilson method).

Several other characteristics improved with increased device use from less than once a week to daily use. Ability to walk long distances and stand for long periods of time improved from 9% and 7% to 19% and 20%, respectively (*P* < .0001). Ease of wearing compression garments also increased from 7% to 21% (*P* < .0001). Finally, patient self-image rose from 1% to 11% (*P* < .0001).

Most patients (61%) reported daily usage of the device, whereas 28% reported 3 to 6 d/wk, 6% reported 1 to 2 d/wk, and 1% reported less than once a week at the 45-day time point (n = 7524).

### Comparing 45- and 90-day outcome

**Fig 4:**
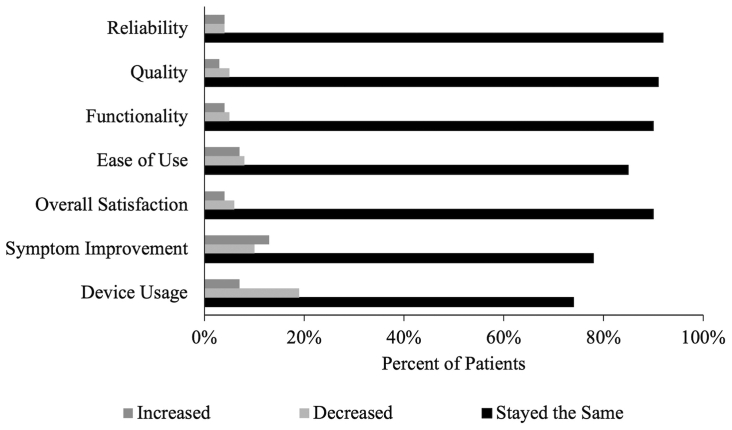
Comparison of changes in patient-reported satisfaction across various categories (reliability, quality, functionality, ease of use, and overall satisfaction), frequency of device usage, and symptom improvement and/or overall ability to perform normal activities between the 45- and 90-day time points (satisfaction categories, n = 3346 patients; device usage, n = 3330 patients; symptom improvement, n = 3267 patients). Confidence intervals are 95% confidence intervals calculated using Wilson method.

Patients were surveyed again at 90 days to assess change in outcomes over time, which were compared with 45 days. Across all satisfaction categories, the responses stayed the same for 85% to 92% of patients (73%-88% of which were already Likert score 4-5), decreased for 4% to 8% and increased for 3% to 7%. For change in how often the device was used, responses stayed the same for 74%, decreased for 19%, and increased for 7% (Fig 4).

For symptom improvement and ability to perform normal activities, responses stayed the same for 78% (56% of which were already Likert score 4-5), decreased for 10%, and increased for 13% (Fig 4). The significant improvement in swelling, heaviness, and pain at 45 days stayed the same for 73%, 75%, and 79%, respectively.

Patients' ability to walk longer distances or stand for longer periods of time stayed the same for 82% and 80%, respectively. Ease of wearing garments was unchanged for most patients (79%). Finally, patient self-image stayed the same for 89%.

## Discussion

This study on population of patients with lower-extremity lymphedema revealed high satisfaction across various categories and significant symptom improvement with PCD usage. Most of the study population was made up of middle-aged to older women with bilateral lower-extremity lymphedema, which aligns with the predominant demographic impacted by lymphedema.^4^^,^^8^^,^^10^ Multiple studies have demonstrated significant benefits to PCD use for lower-extremity lymphedema with improvements in objective outcomes of limb girth, cellulitis, hospitalizations, resource usage, ulcerations, and quality of life.^3^^,^^4^^,^^11^ This study aimed to expand upon this literature with a sole focus on patient-reported outcomes.

Patients reported a high level of satisfaction with the device overall, including its ease of use, functionality, quality, and reliability at both 45 and 90 days. It is critical to assess such subjective patient-reported outcomes because even small changes in objective metrics such as limb volume reduction can result in large changes in subjective quality of life and vice versa.^4^ Other studies using patient-reported metrics to assess advanced PCD outcomes have similarly reported high patient satisfaction rates reaching 96% and significantly higher quality of life scores over 1 year.^3^^,^^4^ Ease of use for PCD therapy was also reported to be high (86%) in a survey-based study on phlebolymphedema.^8^ Given that high satisfaction is a major contributor to high adherence rates, which in turn leads to treatment success, these findings highlight the substantial advantage to incorporating a PCD in longitudinal lymphedema therapy.^8^

Furthermore, patient satisfaction significantly rose with increased frequency of device use from less than once a week (48%-77%) to 3 to 6 d/wk (77%-93%), Likert 4 and 5 combined, incentivizing the frequent usage of PCDs. Most patients in this study used the device daily (61%), compared with the widely ranging adherence rates of 28% to 69% reported in the literature.^6^ However, as frequency of device use rose from to 3 to 6 d/wk (77%-93%) to daily (85%-94%) use, there was minimal improvement observed in patient satisfaction overall (Likert 4 and 5 combined). Although data are limited on optimal dosing and frequency of PCD use, this study finding suggests a potential plateauing of optimal response achievement at 3 to 6 d/wk, which should be explored further.^12^

Patients reported significant symptom improvement and ability to perform daily activities while using the PCD, which improved with increased device usage from less than once a week (34%) to 3 to 6 d/wk (63%) (Likert 4 and 5 combined). Most subjects reported improvement in swelling (58%), heaviness (34%), and pain (25%) with daily use. An observational study assessing patient-centered outcomes (n = 52 patients) with advanced PCD usage for phlebolymphedema also reported significant improvements in swelling (91%) and pain (86%), both major symptoms that would otherwise result in poor function in these patients.^8^ Minimal additional benefit was derived from using the device 3 to 6 d/wk (63%) vs daily (67%).

Patient satisfaction (85%-92%), device usage (74%), and symptom improvement (78%) trends remained the same for the majority of patients when comparing 45- and 90-day time points. Thus, all measured outcomes in this study were achieved by 45 days and were sustained over time.

Limitations of this study include its inherent survey-based design that may introduce response bias into the sample such as toward satisfied users. Given both device usage and outcomes were self-reported, it may introduce susceptibility to common method bias and reverse causation. Data were not available on patients who did not complete the survey, and there may be a degree of nonresponse bias. It was also limited to patients who had active territory account managers and emails, recruiting more engaged patients. Furthermore, it has a 30% response rate although this is in the higher rate of survey returns. The survey itself was also not validated, although it had components similar to others. Statistically, the Likert scale was analyzed by dichotomizing it into 1 to 3 and 4 and 5 to make clinically meaningful comparisons, but it could inadvertently introduce potential inflation of effects. Our outcomes were focused on patient-reported subjective rather than objective measures. Although noted, no direct adjustment was made in this study for factors such as disease severity, etiology, or body mass index, which could be assessed in future studies. Finally, the questionnaire was exclusively administered to outpatients using devices from a single manufacturer.

## Conclusions

Overall, PCD therapy offered safe and substantial benefit in the treatment of lower-extremity lymphedema when evaluated through patient-reported outcomes. It demonstrated a high satisfaction with the device overall, including its ease of use, functionality, quality, and reliability. Symptom improvement, especially of swelling, heaviness, and pain, was also high. Both satisfaction rates and symptom improvement increased with device usage, underscoring the importance of patient adherence to treatment. Considering the time and burden of PCD usage, the observation of therapeutic equipoise achieved with only 3 to 6 d/wk vs daily use is novel and warrants further clinical evaluation. These clinical benefits were achieved as early as 45 days and maintained through 90 days. This study advocates to incorporating a PCD into lymphedema treatment algorithms, expanding of insurance coverage for home-based therapy, and future studies investigating their optimal frequency of usage.

## Author Contributions

Conception and design: NL

Analysis and interpretation: AsR, AlR, SD, AG, NL

Data collection: NL

Writing the article: AsR, AlR, NL

Critical revision of the article: AsR, AlR, SD, AG, NL

Final approval of the article: AsR, AlR, SD, AG, NL

Statistical analysis: AsR, NL

Obtained funding: Not applicable

Overall responsibility: NL

## Funding

None.

## Disclosures

S.D. and A.G. are consultants for Tactile Medical. The remaining authors report no conflicts.
